# On the Effects of Secluded and Gloomy Imprisonment on Individuals of the African Variety of Mankind, in the Production of Disease

**Published:** 1843-11-11

**Authors:** 


### BIBLIOGRAPHICAL NOTICES.

*On the Effects of Secluded and Gloomy Imprisonment on individuals of the African variety of Mankind, in the production of disease.* By Benjamin H. Coates, M. D. Read at the Centennial Anniversary of the American Philosophical Society, May 29th, 1843. Philadelphia: 1843. 8vo. pp. 13.

Whilst an official visiter of the Eastern Penitentiary of this State, Dr. Coates' attention was drawn to the great inequality in the operation of separate and gloomy confinement on individuals of the white and colored races of mankind. From an examination of the different tables collected and published by Dr. C. we find that—

"In 1832 three coloured deaths occurred and only one white; though the whites outnumbered the coloured in the proportion of 69 to 22; rejecting fractions and taking the nearest unit. In 1834, the coloured deaths were four, the whites one; coloured population 59, white 124. Next year the deaths were 5 and 2, though the larger mortality occurred among 108, and the smaller among 155. In 1836, the numbers were 10 and 2, among a population, respectively, of 147 and 202. In 1839, they were 8 and 2, among 173 and 245, respectively. This had not ceased to exist in 1842, when they were 6 and 3, among 130 and 212."

There was no distinction between the treatment of the two races. The increase of death among the coloured prisoners was due, it appears, to scrofula, in its several forms, as we shall presently show. From the statistical researches of Dr. Emerson, we find that, from 1820 to 1831, inclusive, out of an equal number of each complexion residing in our city and suburbs, to every hundred deaths of white persons, one hundred and ninety-six coloured persons died. Now, from the tables published by Dr. Coates, it would appear that, out of an equal number of both complexions residing in the Penitentiary, to every hundred deaths of white persons there would die three hundred and forty-six coloured persons.

No one, in the face of these facts, can doubt the conclusions of Dr. Coates, that "there exists an immense discrepancy in the effect of imprisonment between the coloured people and the whites; and that there is an essential difference in this, as in so many other respects, between the two races."

"Of 43 deaths of coloured convicts, twenty-nine are ascribed to chronic diseases of the lungs, and to affections of an adjacent structure, in which these are extremely liable to produce such diseased changes; and ten to scrofula of other parts than the lungs; leaving four for all other affections; and these four were produced by typhus fever, remittent fever, asthenia, and tetanus. Of nineteen deaths among white persons, on the other hand, the causes are found in chronic diseases of the lungs and their appendages, for eleven cases; scrofula, *none;* chronic bowel complaint, two; small pox, two; and four others are severally attributed to syphilis, asthenic brain fever, stone, and diseased arteries, with enlarged heart. Reduced to per centages, these proportions would read as follows:—

*Coloured.*

| | |
|---|---|
| Diseases of chest, exclusive of heart and arteries, | 65.12 |
| Scrofula, | 23.25 |
| All other diseases, | 11.63 |
| Total, | 100. |

*White.*

| | |
|---|---|
| Diseases of chest, | 57.89 |
| Scrofula, | 0. |
| All other diseases, including bowel complaints, 10.53, and small pox, 10.53, | 42.11 |
| Total, | 100. |

Our own attention has been called to this subject within the past year, from the number of cases of tubercular disease sent to the black wards of the Philadelphia Hospital from the prisons. If a very limited experience justifies any opinion on our part, it is favorable to the views of Dr. Coates.

The thanks of all humane persons are due to Dr. Coates for calling attention to this point; and should the results here stated be confirmed by future inquiries, we trust that the matter will receive that consideration from the proper source to which it is entitled.

